# Natural Products and Neuroprotection

**DOI:** 10.3390/ijms20225570

**Published:** 2019-11-07

**Authors:** Cristina Angeloni, David Vauzour

**Affiliations:** 1 School of Pharmacy, University of Camerino, 62032 Camerino, Italy; 2 Norwich Medical School, University of East Anglia, Norwich NR4 7UQ, UK

Neurodegenerative diseases are among the most serious health problems affecting millions of people worldwide, and their incidence is dramatically growing together with increased lifespan [1]. These diseases are a heterogeneous group of chronic, progressive disorders characterized by the gradual loss of neurons in the central nervous system, which leads to deficits in specific brain functions. The most common neurodegenerative diseases are Alzheimer’s disease (AD), Parkinson’s disease (PD), amyotrophic lateral sclerosis, multiple sclerosis, and Huntington’s disease. While the etiology of most neurodegenerative diseases is mainly unknown, it is largely recognized that these disorders share common molecular and cellular characteristics that contribute to their progression. These include oxidative stress, mitochondrial dysfunction, protein misfolding, excitotoxicity, dysregulation of calcium homeostasis, and inflammation [2,3,4,5]. There are currently no therapeutic approaches to cure or even halt the progression of these disorders, and existing treatments remain largely palliative. In this context, natural products, because of their broad spectrum of pharmacological and biological activities, are considered promising alternatives for the treatment of neurodegeneration as they might play a role in drug development and discovery. A number of studies showed health-promoting properties in the use of natural products as potential therapeutics for neurodegeneration [6,7,8]. Natural compounds have been reported to possess different biological activities, including antioxidant, anti-inflammatory, and antiapoptotic effects [9,10]. Moreover, natural compounds have been recently shown to counteract protein misfolding and to modulate autophagy and proteasome activity [11,12].

The papers published as part of this Special Issue deal with two different forms of natural products: extracts and isolated compounds. The study of the bioactivity of the extracts is extremely important as in vivo natural compounds are usually obtained through the diet as a complex mixture. The importance of extracts is further supported by the fact that many studies have demonstrated the synergistic effect of the combination of different natural products [13]. On the other hand, the investigation of the activity of specifically isolated natural products can be also important to understand their cellular and molecular mechanisms and to define what are the specific bioactive components in extracts or foods.

Research conducted by Sabti M. and colleagues [14] elucidated the molecular mechanisms underlying the relaxant and anxiolytic properties of *Lippia citriodora* (VEE) and verbascoside (Vs), a phenypropanoid glycoside. *Lippia citriodora* is a plant from the Verbenaceae family and is cultivated in North Africa, Southern Europe and the Middle East. In this study both an in vivo mouse model of anxiety and depression and the in vitro SH-SY5Y cell line were employed. In particular the authors evidenced a relaxation effect of high doses of VEE associated with the regulation of genes playing key roles in calcium homeostasis (calcium channels), cyclic AMP (cAMP) production and energy metabolism. Low doses of VEE and Vs showed an antidepressant-like effect by enhancing brain-derived neurotrophic factor (BDNF), noradrenalin, serotonin and dopamine expressions. These results were further confirmed in vitro as both VEE and Vs enhanced cell viability, mitochondrial activity and calcium uptake in SH-SY5Y cells.

In their manuscript, Lee Y.G. et al. [15] isolated four flavonols, three flavones, four flavanonols, and one flavanone from a *Chionanthus retusus* extract, a deciduous tree of the Oleaceae family mainly cultivated in Korea, Japan and China. Eight of these flavonoids demonstrated to be effective in counteracting inflammation by inhibiting nitric oxide (NO) production in RAW 264.7 cells activated by lipopolysaccharide. In addition, these flavonoids showed a neuroprotective activity counteracting glutamate-induced cell toxicity increasing heme oxygenase 1 (HO-1) protein expression in mouse hippocampal HT22 cells.

Similarly, Jang Y. et al. [16] demonstrated that auraptene (AUR), a 7-geranyloxylated coumarin isolated from citrus fruit, is able to counteract neurotoxin-induced reduction of mitochondrial respiration and to inhibit reactive oxygen species (ROS) generation in SN4741 mouse embryonic substantia nigra dopaminergic neuronal cell line. Moreover, they observed, in a MPTP-induced PD mouse model, that AUR treatment improved movement deficits in association with an increase in the number of dopaminergic neurons in the substantia nigra.

Chiroma S.M. et al. [17] investigated the neuroprotective effect of *Centella asiatica* (CA), a plant from the family of Apiaceae, in a rat model of neurodegeneration induced by d-galactose/aluminum chloride (d-gal/AlCl3). These authors previously observed that CA extract can attenuate cognitive deficits in this model of neurodegeneration and can also prevent morphological aberrations in the CA1 region of hippocampus [18]. In the paper published in this Special Issue, they demonstrated that CA significantly increased the levels of protein phosphatase 2 and decreased the levels of glycogen synthase kinase-3 beta. Moreover, CA extract also counteracted apoptosis as it increased the expression of the Bcl-2 mRNA level.

Finally, Javed H. et al. [19] demonstrated the neuroprotective effect of thymol, a dietary monoterpene phenol, in a rat model of PD. In particular, neurodegeneration was induced by rotenone at a dose of 2.5 mg/kg body weight for four weeks. Thymol, co-administered to rotenone for four weeks at a dose of 50 mg/kg body weight, significantly attenuated dopaminergic neuronal loss, oxidative stress and inflammation suggesting a protective effect of thymol in rotenone-induced PD.

Along with research papers, different reviews are also presented in this Special Issue.

As previously underlined, proteostasis failure plays a crucial role in the context of ageing and neurodegeneration. Therefore, natural products targeting the proteostasis elements emerge as a promising neuroprotective therapeutic approach to prevent or ameliorate the progression of these disorders. Cuanalo-Contreras K. et al. [20] focused on this aspect and revised the current knowledge regarding the use of natural products as modulators of different components of the proteostasis machinery to counteract neurodegeneration. The majority of natural modulators of the proteostasis network are of plant-origin, however some compounds of marine-animal-origin are also emerging. They concluded that further studies are required to understand the precise mechanism of action of the natural proteostasis activators, their off-target effects and their in vivo bioavailability. In their review, Cho B. et al. [21] focused on the effect on natural products on the proteostasis elements such as ubiquitin‒proteasome system and autophagy (mitophagy) in experimental PD models. Moreover, in the same experimental models, they also revised the neuroprotective effects of natural products on mitochondrial dysfunction, oxidative stress, and hormesis. They summarized the efforts to use natural extracts as lead compounds for the design of novel pharmacological candidates for the treatment of age-related PD. Finally, they addressed two main limitations in the use of natural compounds in counteracting neurodegeneration: the differences of experimental design, such as the quality of the extracts and the forms of dosage, of the studies and the unclear therapeutic mechanism of natural compounds.

Taking into account these two limitations Di Paolo M. et al. [22] analyzed the ethical framework of the potential clinical use of natural products to counteract neurodegeneration, with particular attention paid to the principles of biomedical ethics. They concluded that natural products could represent a great promise for the treatment of neurodegeneration, where traditional therapies, via synthetic drugs, only act to alleviate symptoms. However, lack of knowledge on the efficacy and safety of many natural products underscores the urgent need for further investigation to better characterize the therapeutic mechanism of natural products in order to promote patient safety and ethical care.

Park J.Y. et al. [23] revised the current research on the structural diversity, biosynthesis, and pleiotropic neuronal functions of ascaroside (ascr) pheromones and their implications in animal physiology. Pheromones are neuronal signals that stimulate conspecific individuals to react to environmental stressors or stimuli. The authors also discuss the concentration and stage-dependent pleiotropic neuronal functions of ascr pheromones. They suggest that in the future, translation of the knowledge of nematode ascr pheromones to higher animals might be beneficial, as it has been observed that ascr has some anti-inflammatory effects in mice.

Pervin M. et al. [24] discuss the function of (−)-epigallocatechin gallate (EGCG) and its microbial ring-fission metabolites in the brain as neuroprotective agent. EGCG, the main green tea catechin, is an ester of (−)-epigallocatechin (EGC) and gallic acid (GA). Despite the great number of studies on the neuroprotective effects of green tea catechins against neurological disorders, it should take into account that the concentration of EGCG in systemic circulation is very low and EGCG disappears within several hours. EGCG undergoes extensive metabolism and recent studies suggest that metabolites of EGCG may play an important role, alongside the beneficial activities of EGCG, in reducing neurodegenerative diseases.

Barbalace M.C. et al. [25] focused on the effect of marine algae on neuroinflammation, one of the main contributors to the onset and progression of neurodegenerative diseases. As pointed out by Cuanalo-Contreras K. et al., marine organisms represent a vast source of natural compounds, and among them, algae are an appreciated source of important bioactive components. Barbalace et al. revised the numerous anti-inflammatory compounds that have been recently isolated from marine algae with potential protective efficacy against neuroinflammation.

Polyphenols are among the most studied dietary molecules probably for their multiple and often overlapping reported modes of action. Epidemiological studies suggest a strong association between polyphenol consumption and reduced prevalence of various neurodegenerative diseases; however, ambiguity still exists as to the significance of their influence on human health. Renaud J. and Martinoli M.G. [26] analyzed the characteristics and functions of polyphenols that determine their potential therapeutic actions in neurodegenerative disorders. In particular, they discuss the properties that may influence the functionality and bioavailability of dietary polyphenols in the central nervous system (CNS) with a particular focus on therapeutic applications and limitations.

Among polyphenols, curcumin, a component of *Curcuma longa*, is currently considered one of the most effective nutritional antioxidants due to its activity in multiple antioxidant and anti-inflammatory pathways involved in neurodegeneration. Mhillaj E. et al. [27] provides a summary of the main findings involving the heme oxygenase/biliverdin reductase system as a valid target in mediating the potential neuroprotective properties of curcumin. Moreover, they address the pharmacokinetic properties and concerns about curcumin’s safety profile.

Maher P. [27] focused on a wide class of polyphenols, flavonoids. Among the huge number of polyphenols, several epidemiological studies have specifically highlighted the potential beneficial role of flavonoids to counteract neurodegeneration. In particular the author discusses the beneficial effects of multiple flavonoids in different models of neurodegenerative diseases and identified common mechanisms of action. As outlined by other authors of this Special Issue, the conclusions state that further investigations should be carried out in order to use flavonoids in the treatment of neurodegenerative diseases.

Infante-Garcia C. and Garcia-Alloza M. [28] reviewed natural compounds with a protective activity against brain neurodegeneration in animal models of diabetes mellitus, taking into account several therapeutic targets: inflammation and oxidative stress, vascular damage, neuronal loss or cognitive impairment. Diabetic brain is characterized by micro and macrostructural changes, such as neurovascular deterioration or neuroinflammation that lead to neurodegeneration and progressive cognition dysfunction. The authors evidenced that natural compounds and extracts show antioxidant and anti-inflammatory activities at a central level, as well as a relevant capacity to reduce vascular damage, contributing altogether to limit neurodegeneration and cognitive derived alterations. In their conclusion the authors highlighted that natural products could contribute to expand therapeutic options to treat or reduce central complications associated with diabetes mellitus.

Andrade S. et al. [29] focus their attention on a specific neurodegenerative disease, AD, and discuss both the natural compounds already in clinical trial phase and other natural compounds with known potentially beneficial effects in AD in a preclinical development stage. Regarding the preclinical studies, only the most recent reported works have been considered. Clinical trials have demonstrated that different compounds appear to be effective for AD therapy, on the contrary others have failed in human trials. Natural compounds in earlier phases of research need further studies to uncover their therapeutic potential for AD.

Berezowska M. et al. [21] reviewed the effects of vitamin D in multiple sclerosis on pathology and symptoms. Based on specific criteria, they selected ten studies with a size ranging from 40 to 94 people and with a duration of the intervention from 12 to 96 weeks; all the studies compared the use of vitamin D with a placebo or low dose vitamin D. One trial found a significant effect on Expanded Disability Status Scale (EDSS) score, three demonstrated a significant change in serum cytokines level, one found benefits in enhancing lesions and, interestingly, three studies reported no serious adverse events in the use of vitamin D.

In conclusion, the papers published in this Special Issue, despite addressing different topics, can be considered an important contribution to the knowledge of the neuroprotective effect of natural products, and present a great deal of information related to both the benefits but also the limitations of their use in counteracting neurodegeneration.
